# Identification of area-level influences on regions of high cancer incidence in Queensland, Australia: a classification tree approach

**DOI:** 10.1186/1471-2407-11-311

**Published:** 2011-07-24

**Authors:** Susanna M Cramb, Kerrie L Mengersen, Peter D Baade

**Affiliations:** 1 Viertel Centre for Research in Cancer Control, Cancer Council Queensland, Gregory Tce, Fortitude Valley, Australia; 2 Centre for Data Analysis, Modelling and Computation, Queensland University of Technology, George St, Brisbane, Australia; 3 School of Public Health, Queensland University of Technology, Herston Rd, Kelvin Grove, Australia

**Keywords:** cancer incidence, socioeconomic factors, indigenous population, rural health, classification and regression tree

## Abstract

**Background:**

Strategies for cancer reduction and management are targeted at both individual and area levels. Area-level strategies require careful understanding of geographic differences in cancer incidence, in particular the association with factors such as socioeconomic status, ethnicity and accessibility. This study aimed to identify the complex interplay of area-level factors associated with high area-specific incidence of Australian priority cancers using a classification and regression tree (CART) approach.

**Methods:**

Area-specific smoothed standardised incidence ratios were estimated for priority-area cancers across 478 statistical local areas in Queensland, Australia (1998-2007, n = 186,075). For those cancers with significant spatial variation, CART models were used to identify whether area-level accessibility, socioeconomic status and ethnicity were associated with high area-specific incidence.

**Results:**

The accessibility of a person's residence had the most consistent association with the risk of cancer diagnosis across the specific cancers. Many cancers were likely to have high incidence in more urban areas, although male lung cancer and cervical cancer tended to have high incidence in more remote areas. The impact of socioeconomic status and ethnicity on these associations differed by type of cancer.

**Conclusions:**

These results highlight the complex interactions between accessibility, socioeconomic status and ethnicity in determining cancer incidence risk.

## Background

Globally, almost 12.7 million people were diagnosed with cancer in 2008 (excluding non-melanoma skin cancers), and 7.6 million people died from cancer [1]. Cancer was the third highest cause of death (following cardiovascular disease and infectious and parasitic diseases) [2].

In Australia, cancer was responsible for almost 40,000 deaths and 108,368 diagnoses (again, excluding non-melanoma skin cancer) in 2007 [3]. Cancer was estimated to be the greatest contributor to the burden of disease, causing 19% of the entire disease burden, and half of this was due to lung, colorectal, prostate and breast cancers [3]. Due to its high morbidity and mortality, cancer is an Australian government health priority area, with specific emphasis placed on the National Health Priority Area (NHPA) cancers of colorectal cancer, lung cancer, melanoma, non-melanoma skin cancer, breast cancer, cervical cancer, prostate cancer and non-Hodgkin's lymphoma [4].

Government strategies for cancer reduction and management are targeted at both the individual and area levels. Recognised risk factors at the individual level for cancer incidence include tobacco smoke exposure, ultraviolet exposure, diet, exercise and genetics [5]. Evidence is accumulating that area-level effects, such as socioeconomic inequality, ethnic composition, civic engagement, government policies and accessibility can shape many of the individual risk factors [6]. Area-level strategies require careful understanding of geographic differences in cancer incidence, in particular the association with factors such as socioeconomic status, ethnicity and accessibility. These factors are not independent, since rural and remote regions of Australia are more likely to be of lower socio-economic status, and similarly urban areas are more likely to have higher socio-economic status [7].

This study aimed to identify the complex interplay of area-level factors associated with areas of high incidence of the Australian priority cancers, and through this demonstrate the application of classification and regression trees (CART) for this purpose. Unlike more traditional regression models, CART models are able to identify interactions between ecological factors that best split geographical areas into homogenous subgroups based on their relative incidence rates.

## Methods

Incidence data for the NHPA cancers (excluding non-melanoma skin cancer) covering the period 1998-2007 were obtained from the Queensland Cancer Registry (QCR) after obtaining approval from Queensland Health (Ethics approval number: HREC/09/QHC/25). The QCR is a population-based registry, which maintains a record of all cancer cases (excluding non-melanoma skin cancer) diagnosed in Queensland since 1982, and to which notification is required by law [8]. Cancers were classified according to the World Health Organization's International Classification of Diseases for Oncology, 3^rd ^edition (ICD-O3). Population estimates were obtained from the Australian Bureau of Statistics (ABS) [9,10].

The geographic regions used for this analysis are Statistical Local Areas (SLAs) which cover Queensland without gap or overlap. In 2006 there were 478 SLAs, ranging in population size from 7 to 77,523, with a median population of 5,810. SLAs were categorised by accessibility, socio-economic status and Indigenous composition. Accessibility was defined by the Accessibility/Remoteness Index of Australia (ARIA+), which categorises areas as 'Major Cities (MC)', 'Inner Regional (IR)', 'Outer Regional (OR)', 'Remote (R)' or 'Very Remote (VR)' [11]. These categories are determined by the minimum road distance from population localities to different levels of service centres [11]. Socioeconomic status was defined using the Socioeconomic Indexes for Areas (SEIFA) Index of Relative Socioeconomic Disadvantage (IRSD) [12]. SLAs in Queensland were ranked from the most disadvantaged to the least disadvantaged and then divided into quintiles. For clarity we refer to the quintiles as 'Most Disadvantaged (MD)', 'Moderately Disadvantaged (ModD)', 'Middle SES (MSES)', 'Moderately Advantaged (ModA)' and 'Most Advantaged (MA)'. For ease of reference, 'advantaged' areas include 'most advantaged' and 'moderately advantaged', and similarly for 'disadvantaged' areas. SLAs were considered to be Indigenous if at least 10% of the population identified as Aboriginal or Torres Strait Islander in the 2006 population census [13].

The data analysis comprised four main steps: (i) estimating smoothed Standardised Incidence Ratios (SIRs) for each cancer; (ii) identifying cancers with significant spatial variation; (iii) identifying SLAs with "high" incidence for each cancer, based on the smoothed SIR estimates, and (iv) for these cancers, identifying the area-level factors associated with high incidence SLAs.

For Step (i), incidence data were adjusted for age by indirect standardization to provide empirical SIRs by cancer type and gender. A Bayesian hierarchical spatial smoothing model (known as the Besag, York and Mollié model) was then applied to produce smoothed SIRs [14]. This model assumes that neighbouring SLAs should be more similar than SLAs further away, with respect to the SIR values (or the associated factors, such as accessibility, socio-economic status and ethnicity). Thus smoothed SIR estimates are to some extent averaged over neighbouring values; this also helps address the problem of unstable empirical estimates that are based on small population sizes [15]. The model was run using Stata interfaced with WinBUGS [16]. Further details regarding the methodology are described elsewhere [17].

We restricted the detailed analyses to those cancers that had significant sex-specific area-level variation, or heterogeneity, in the smoothed SIR estimates (Step (ii)). This area-level variation was assessed using the Tango's Maximised Excess Events Test (MEET) [18]. Values of Tango's MEET that were < 0.05 were deemed to reflect statistically significant variation in estimates.

For Step (iii), the smoothed SIR estimates were classified as 'high' if they were at least 10% greater than the Queensland average. Sensitivity analyses examining the influence of alternate cutpoints (5% and 15% above the Queensland average) were also conducted.

For Step (iv), a weighted CART model was fitted for each of the cancers selected in Step (ii). The aim of the CART model is to identify a sequence of binary splits of the area-level factors (accessibility, socioeconomic status, ethnicity) that best divide the high/not high smoothed SIRs for each SLA into homogeneous subgroups. The resultant sequence of splits resembles a tree-like structure, and the final subgroups are known as 'terminal nodes' that can be described as high if the estimated Pr(high SIR) is greater than 0.5. The best tree was chosen using the minimum cross-validation criterion, which chooses the tree with the lowest expected error if new data were to be applied to this model (cross-validated error) [19]. In all cases this gave the same result as using the alternative one-standard-error rule, which is calculated as the tree with the fewest nodes which has a cross-validated error below the sum of the minimum cross-validated error and its standard error [19]. The CART analysis was conducted using the RPART package in R version 2.11.1 [20]. Annotated code is provided in the Appendix. To adjust for differences in the precision of the smoothed SLA-specific estimates, the inverse of the variance was used to weight the dichotomous SIR variable.

The sensitivity and specificity for each final tree was also calculated. Sensitivity was the weighted sum of true positive values divided by the weighted sum of false negative values. Similarly, specificity was calculated as the weighted sum of false positive values divided by the weighted sum of true negative values.

In the CART diagrams, the terminal nodes are portrayed by rectangles. Within each terminal node (or rectangle) are three rows of numbers. The first contains the number of SLAs with a high SIR value versus the total number of SLAs in the node. The second row contains the Pr(H) value, which is the weighted proportion of SLAs with a high SIR in the subgroup of SLAs represented in the node. The third row contains the 95% confidence interval (CI) for the probability of a high SIR, calculated as  where *p *is the Pr(H) and *n *is the number of SLAs. In the few instances where a CI value surpassed the possible (0,1) boundaries, this was restricted to the appropriate boundary value. The CART diagrams are also accompanied by summary diagrams showing which areas were likely to have high SIR values (shaded as dark grey), and which were likely to not have high SIR values (shaded as light grey). These contain ARIA and SEIFA combinations to facilitate comparison between cancer types. Combinations which do not exist were rendered in white. Note the same shading is also used for the terminal nodes in the CART diagram. Dark grey terminal nodes are likely to have a high SIR, in contrast to the light grey terminal nodes.

## Results

The cancers that had statistically significant evidence of variation in the smoothed SIR estimates were lung cancer, melanoma, breast cancer (females), cervical cancer, prostate cancer, and non-Hodgkin lymphoma (Table 1). There was no significant evidence of geographical variation in colorectal cancer incidence for males (p = 0.693) or females (p = 0.216). The sensitivity of the final CART models ranged from 51.5% (female lung cancer) to 97.2% (female non-Hodgkin lymphoma), while the specificity ranged from 31.1% (female melanoma) to 82.7% (female lung cancer) (Table 1).

**Table 1:** Summary of area-level variation for National Health Priority Area cancers and CART analysis results

Type of cancer (ICD-O3 code)	Gender	Tango's MEET	Number of SLAs with high SIR (%)	Sensitivity	Specificity	Variables in final tree^1,2^
Colorectal cancer	M	0.693	NA	NA	NA	NA
(C18-C20, C218)	F	0.216	NA	NA	NA	NA
Lung (C33-C34)	M	0.001	153 (32%)	70.1%	74.9%	SEIFA, ARIA
	F	0.001	83 (17%)	51.5%	82.7%	ARIA, SEIFA
Melanoma	M	0.001	91 (19%)	75.0%	49.8%	ARIA
(C44 and M872-M879)	F	0.004	54 (11%)	93.7%	31.1%	ARIA, SEIFA
Breast (C50)	F	0.001	79 (17%)	86.5%	58.1%	ARIA, SEIFA
Cervical (C53)	F	0.023	81 (17%)	79.2%	79.3%	ARIA, I, SEIFA
Prostate (C61)	M	0.001	93 (19%)	70.1%	58.5%	ARIA, SEIFA
Non-Hodgkin's lymphoma	M	0.001	57 (12%)	90.1%	38.7%	ARIA
(M959,M967-M971)	F	0.002	57 (12%)	97.2%	55.2%	ARIA

1. The final tree based on the lowest cross-validated error.

2. NA: since there was no evidence of area-level variation for colorectal cancer, additional analysis was not conducted for colorectal cancer.

ARIA: Accessibility/Remoteness index of Australia

I: Indigenous

SEIFA: Socioeconomic indexes for areas

### Lung cancer

**Figure 1:**
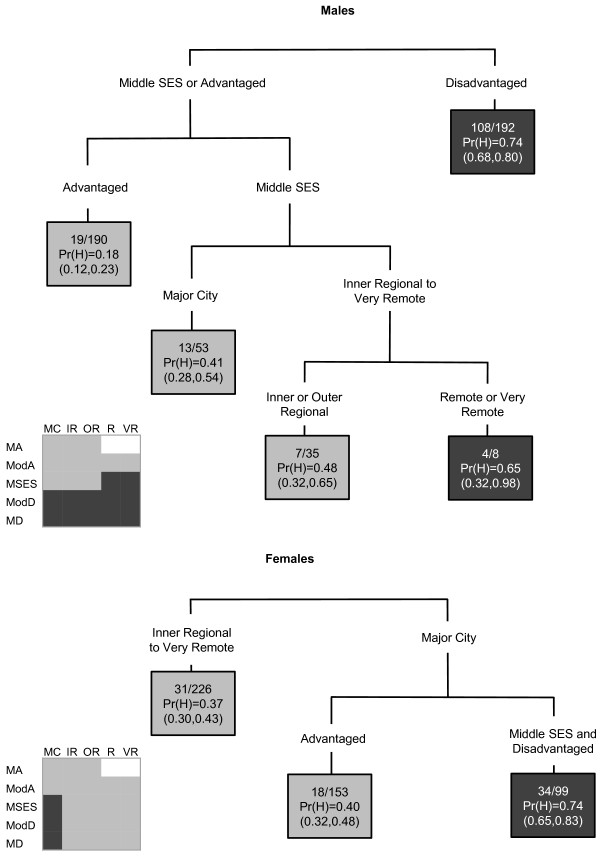
**The final classification and regression tree for lung cancer**.

For lung cancer among males, socioeconomic status was the primary determinant, whereas for females it was the accessibility of an area (Figure 1). There were interactions between socioeconomic status and accessibility for both genders. Areas were more likely to have increased lung cancer incidence among males if they were disadvantaged or were remote and very remote areas of middle SES. Areas within major cities of middle or disadvantaged SES were likely to have a high incidence of lung cancer among females.

### Melanoma

**Figure 2:**
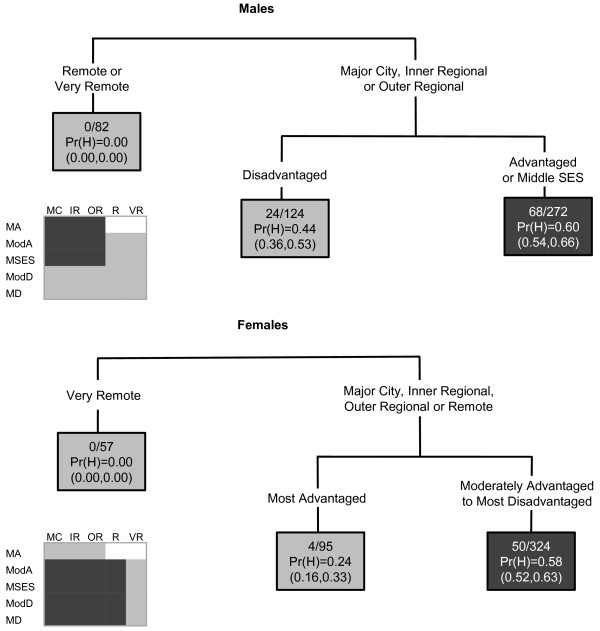
**The final classification and regression tree for melanoma**.

Contrasting patterns were observed for melanoma incidence among males and females. Among males, an area was likely to have a high melanoma incidence if it was classified as a major city, inner or outer regional area and of middle or advantaged SES (Figure 2). In contrast, for females, incidence was higher in all areas except those within the most advantaged quintile, and the very remote areas. Therefore areas of disadvantage were likely to have high incidence among females, but low incidence among males.

### Female breast cancer

**Figure 3:**
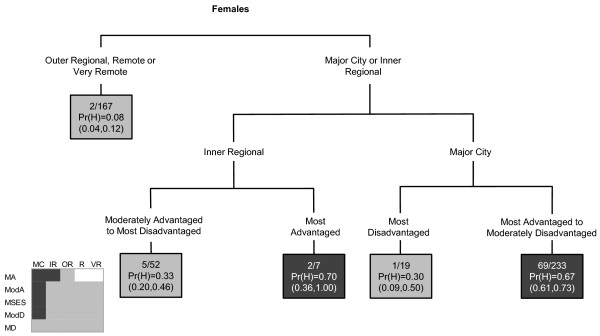
**The final classification and regression tree for breast cancer**.

Breast cancer incidence was likely to be high in areas within major cities, except those that were most disadvantaged. Inner regional areas that were most advantaged were also likely to have high incidence (Figure 3).

### Cervical cancer

**Figure 4:**
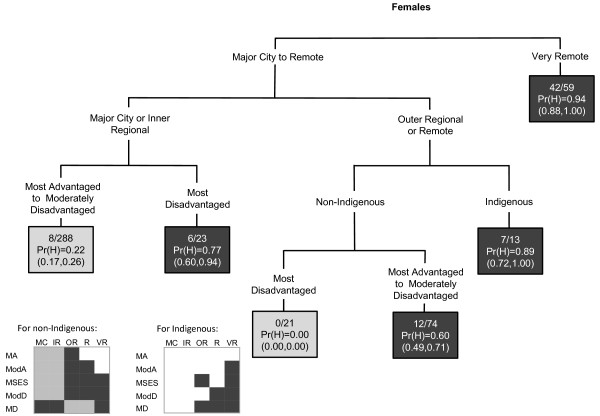
**The final classification and regression tree for cervical cancer**.

Areas that had the highest probability of having increased cervical cancer incidence were those that were most disadvantaged or were in outer regional, remote or very remote areas (Figure 4). However there was also interaction in areas with high Indigenous population; areas that were most disadvantaged, were in outer regional or remote areas and also had a low Indigenous population were more likely to not have a high cervical cancer incidence. Corresponding areas with a high Indigenous population were likely to have a high cervical cancer incidence.

### Prostate cancer

**Figure 5:**
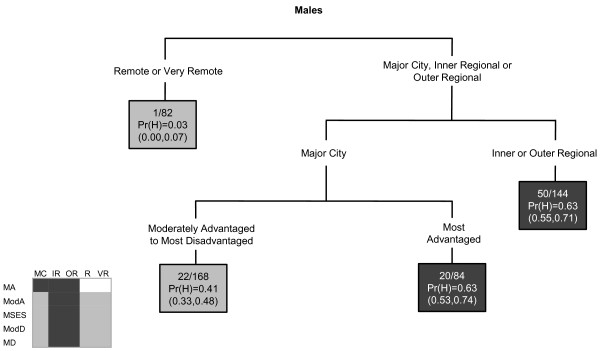
**The final classification and regression tree for prostate cancer**.

Inner and outer regional areas, as well as the socioeconomically most advantaged areas within major cities were likely to have high incidence of prostate cancer among males (Figure 5).

### Non-Hodgkin's lymphoma

**Figure 6:**
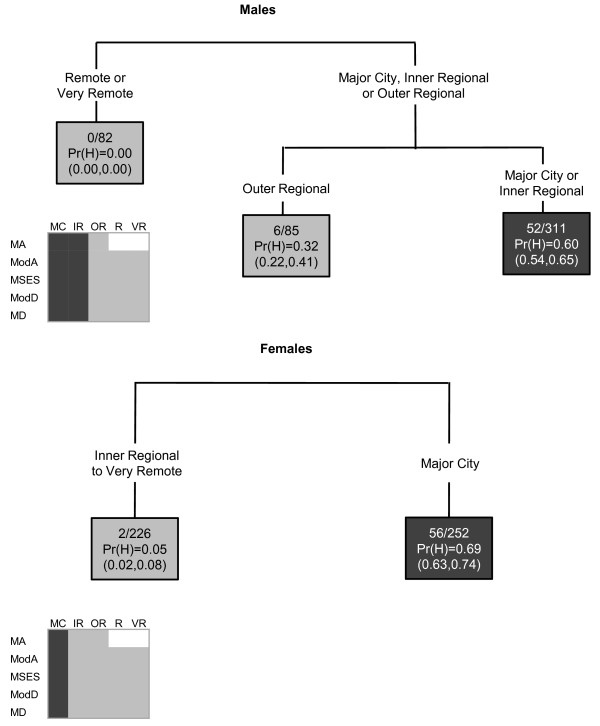
**The final classification and regression tree for non-Hodgkin's lymphoma**.

High incidence of non-Hodgkin's lymphoma was likely to occur among males in major cities or inner regional areas, and among females in major cities (Figure 6).

## Discussion

The accessibility of a person's residence was the greatest predictor of an increased risk of cancer diagnosis across a range of cancers, including lung (females), melanoma, breast (females), cervical, prostate, and non-Hodgkin's lymphoma. Socioeconomic status was the greatest primary explanatory variable for lung cancer (males).

More remote areas had a greater probability of having high incidence of lung cancer among males, and cervical cancer. Cancers for which more urban areas were more likely to have high incidence included: lung cancer (females), melanoma, breast cancer, prostate cancer, and non-Hodgkin's lymphoma.

The interaction between accessibility, socioeconomic status and ethnicity varied depending on the type of cancer. The socioeconomic status interacted with accessibility for lung, melanoma, breast (females), cervical, and prostate cancers. The incidence of cancers that were often screen detected such as breast cancer (females), melanoma (males) and to a lesser extent prostate cancer tended to be higher in more affluent areas, and also more urban areas. In contrast, for lung, melanoma (females) and cervical cancer the incidence was higher in more disadvantaged areas. Cancers with a high incidence in disadvantaged areas did not have a consistent interaction with accessibility. Some tended to be higher in more urban areas (such as lung cancer (females) and melanoma (females)), while others were higher in more remote areas (lung cancer (males) and cervical cancer). Ethnicity also interacted with these factors for cervical cancer, with Indigenous areas more likely to have high incidence.

These results are consistent with previous studies showing an increased incidence of cervical cancers among Indigenous women [21], and an increased incidence of breast cancer among women in more urban or affluent areas [22]. However, there are also important differences compared to previous research. Melanoma incidence has generally been found to be higher in more affluent areas [23]. In contrast, our results found females in the most advantaged areas were less likely to have high incidence, while all other SLAs (except for very remote) were more likely to have high incidence. Queensland has among the highest rates of melanoma in the world [3,24], and this may be impacting on these differences. Similarly, lung cancer incidence has previously been shown to be higher in remote areas for both males and females [25]. However, our results found high incidence among females in the lower socioeconomic areas of major cities.

Individual risk factors could be influencing these geographic differentials. Lung cancer incidence is strongly determined by smoking prevalence 20-30 years earlier [26]. Tobacco smoking has been shown to be more prevalent in lower SES or more remote areas, which may explain the high incidence observed in these areas [27-32]. Similarly, women in affluent areas are more likely to delay childbearing, have fewer children and/or use hormone replacement therapy, all of which are risk factors for breast cancer [33-35].

Preventive measures can also differ geographically. The leading cause of cervical cancer is infection with sexually transmitted human papillomaviruses. Papanicolaou screening (commonly called pap smear testing) detects precancerous lesions, which can then be treated, averting cancer and thus lowering incidence. The high incidence observed in very remote, Indigenous or the most disadvantaged urban areas may result from lower uptake of pap smears. Participation rates for cervical cancer screening (papanicolaou screening) are lower in remote communities and areas of low socioeconomic status in Queensland and throughout Australia [36,37].

In contrast, screening for asymptomatic cancers, such as prostate or breast cancer, can be associated with increased incidence. Therefore access to screening or diagnostic services is another factor which influences incidence and can vary by area. For instance, the incidence of prostate cancer may be inflated in areas where prostate-specific antigen (PSA) testing, which is used to detect asymptomatic prostate cancer, is commonly used. PSA testing is less common in more rural areas than in capital cities throughout Australia [38], and this could be contributing to the lower incidence in remote areas. Breast cancer may also be influenced by geographic variation in screening services, as there is variation in mammogram uptake by accessibility and socioeconomic status [39]. Similarly, the ease of access to skin cancer checking services in more urban areas may influence the incidence of melanoma.

Strengths of the study include the use of routinely collected incidence data from a population-based registry to which notification of cancer is required by law. Queensland has the most decentralized population in Australia [40], thus providing a unique opportunity to investigate these area-based differences in greater detail.

Limitations of the study include the nature of cancer, which takes years to develop and be diagnosed. Therefore it is possible that the incidence of an area may reflect the risk factor prevalence from years earlier, rather than the current situation. Also, estimates were calculated based on area of residence at diagnosis. People may have migrated to different areas leading up to their cancer diagnosis, and any carcinogenic exposure or other area-level influences may have occurred at a different location to where they were diagnosed.

The CART analysis was weighted by the inverse of the variance, which had the effect of placing greater priority on correctly identifying SLAs with high SIRs (or sensitivity), so the specificity (correct identification of SLAs with non-high SIRs) was found to vary considerably between cancers and gender. Two cancers with comparatively low sensitivity and specificity were prostate cancer and male melanoma. Therefore, results for these models should be treated with caution.

The 'high' SIR values were classified as an arbitrary cut-off of at least 10% above the Queensland average. This value was chosen to increase the probability that results were truly above the State average values. Since it was probable that choosing alternate cut-off values would influence the tree structure, sensitivity analyses (not shown) were performed under alternate cut-offs (5% and 15% above the Queensland average). Although different cut-off values often induced some variation in tree structure, the primary split remained identical for all cancers except for minor differences in the categories included on either side of the split for male lung cancer, female breast cancer, cervical cancer, prostate cancer and male non-Hodgkin's lymphoma.

Since the incidence of some cancers such as breast, melanoma and prostate is strongly influenced by screening practices, high incidence may result from overdiagnosis, where asymptomatic cancers are detected which would not otherwise have progressed to cause morbidity and/or death. While in this case a high incidence of cancers may not necessarily be an adverse outcome in itself, the morbidity associated with subsequent treatment is sometimes considerable [41]. Similarly, low incidence may not necessarily be beneficial if the cancers which are diagnosed are detected at a more advanced stage and therefore have worse prognosis. Consistent with other Australian Cancer Registries, the QCR does not routinely collect staging information for all cancers. Therefore it was not possible to differentiate between areas at high risk of having advanced cancers diagnosed, and those at high risk of having sub-clinical cancers diagnosed.

Alternative methods are available to explore interactions. For instance, increasingly cancers are jointly modelled, either using multivariate structures on the relative risks, or latent class models [42]. One benefit of these methods is utilizing strength between the cancers to produce more efficient estimates [43]. By exploring spatial variation in common risk factors, latent class models can provide stronger evidence of any true clustering in the underlying risk surface [43]. However, under latent class joint modeling the shared components (risk factors) for each cancer are pre-specified, whereas the CART analysis determines which of the risk factors are relevant for that cancer. The use of different modelling strategies may identify different features of the data that can lead to better understanding of the problem at hand and can thus lead to more informed inference. For example, in addition to being a valid approach in its own right, a CART model may identify useful interactions for inclusion in a subsequent (univariate or multivariate) regression analysis.

## Conclusions

Identifying which area-level factors are associated with increased incidence enables targeting of resources as well as focusing further exploration for the underlying reasons. This study showed that the accessibility of an area was the main predictor of high incidence for most cancers examined. More often it was the more urban areas which had high cancer incidence, although notable exceptions were cervical and lung cancers (males). In addition, many cancers experienced interaction of the area-level effects, particularly between accessibility and socioeconomic status. These findings highlight the importance of conducting further research exploring the potentially complex reasons underlying these geographical inequalities.

## Appendix

R code used for the CART model:

library(rpart)

#grow the classification tree

fit<- rpart(fail ~ accessibility + socioeconomic + indigenous, weight = weight, method="class", parms = list(prior = c(.5,.5), split='information'), data = data, cp = 0.0001)

printcp(fit) # display the results

plotcp(fit) # visualize cross-validation results

summary(fit) # detailed summary of splits

# plot tree

plot(fit, uniform = TRUE, main="Classification Tree")

text(fit, use.n = TRUE, all = TRUE, cex=.8)

# prune the tree

pfit<- prune(fit, cp = fit$cptable[which.min(fit$cptable[, "xerror"]), "CP"])

# plot the pruned tree

plot(pfit, uniform = TRUE, main="Pruned Classification Tree")

text(pfit, use.n = TRUE, all = TRUE, cex=.8)

## Competing interests

The authors declare that they have no competing interests.

## Authors' contributions

KLM conceived the study. SMC performed the analysis. SMC and PDB drafted the manuscript. All authors contributed to, read and approved the final manuscript.

## Pre-publication history

The pre-publication history for this paper can be accessed here:

http://www.biomedcentral.com/1471-2407/11/311/prepub
